# Editorial: Each child with ADHD is unique: Treat the whole patient, not just their symptoms

**DOI:** 10.3389/fnbeh.2022.1041865

**Published:** 2022-10-04

**Authors:** Edmund J. S. Sonuga-Barke, Salman Zubedat, Essam Daod, Iris Manor

**Affiliations:** ^1^School of Academic Psychiatry, Institute of Psychiatry, Psychology and Neuroscience, King's College London, London, United Kingdom; ^2^School of Medicine, Aarhus University, Aarhus, Denmark; ^3^The Rappaport Faculty of Medicine, Technion-Israel Institute of Technology, Haifa, Israel; ^4^Clalit Health Services, Haifa, Israel; ^5^Geha MHC, Petah Tikva, Israel; ^6^Sackler School of Medicine, Tel Aviv University, Tel Aviv-Yafo, Israel

**Keywords:** ADHD, neurodevelopment, neurodevelopmental disorders, neurogenetics, behavioral and psychiatric genetics

Over the last fifty years extraordinary scientific progress has been attention-deficit/hyperactivity disorder (ADHD) science on a number of levels. First, finding the best way to characterize the condition. Second, understanding of why some are affected by the condition and others in terms of originating environmental and genetic risk, underlying mediating biological and cognitive processes and moderating environments. Third, the awareness of the breadth of ADHD's impact on people's lives (those affected, their families and communities) (see Sonuga-Barke et al., 2023 for a review). However, despite these advances little has changed in terms of how ADHD is managed clinically for a very long time. This means that despite the long-standing availability of medications that give efficacious symptom control, at least in the short term (Young et al., 2021), many people with ADHD continue to suffer considerable disability and disadvantage across their lives (Klassen et al., 2004)—there remains a substantial gap between clinical need and treatment provision.

While speculation continues in the field as to the reason for this disconnect between scientific and clinical progress this *Special Issue* focuses on the notion that the marked heterogeneity seen within the community of people with ADHD, manifest at multiple levels, is one major barrier to translating our science into clinical progress. It goes without saying and is self-evident that clinically ADHD presents symptomatically in different ways in different people—each showing a particular profile of inattention, hyperactivity and impulsiveness. Indeed, this form of heterogeneity is represented in diagnostic systems such as the DSM and ICD adoption of polythetic approaches and the existence of different clinical sub-types or, as they are now termed, presentations. In the case of clinical symptoms these diverse manifestations have common elements and tap shared latent constructs (Wang et al., 2022). In this special issue we focus on two other types of heterogeneity.

The first sort of heterogeneity addressed in this Special Issue relates to variation in the underlying causal processes creating the risk for ADHD—or what has been called “deep heterogeneity” (Sonuga-Barke, 2016). This form of heterogeneity is encapsulated by the first part of the special issue title – ***Each child with ADHD is unique***. From our point of view, this form of pathophysiological heterogeneity represents a major barrier to progress in ADHD translational science. In consequence it represents a significant impediment to therapeutic innovation and improvement. This is likely related to the way, in previous decades, that ADHD science has often been built on the erroneous assumption that ADHD is a singular neuro-biological entity and that people with ADHD are a homogeneous group, sharing a common pathophysiology making them differ from the rest of the population in kind rather than degree. This set of assumptions, which together makes up what philosophers of science term a meta-theory, can be directly sourced back to the concepts of disorder as harmful dysfunction that underpins diagnostic manuals such as the DSM and ICD. This assumption constrains the questions posed by scientists, the methodological approach they employ, and the way results are interpretated. For instance, the pivotal question in ADHD neuroscience has typically been – *Where in the brains (which region, which circuit or network) of the ADHD child is the site of the dysfunction that causes ADHD?* However, it's become increasingly clear from the start of the 21st century that this is a profound mischaracterisation of ADHD. Furthermore, the question which derives from it is a scientific form of a “red herring” (Sonuga-Barke and Castellanos, 2005). This is because it is now indisputable that ADHD is a highly heterogeneous condition, not only clinically (as discussed above) but also in terms of underlying causes and pathophysiology (Balogh et al., 2022; Loh et al., 2022). In this sense, ADHD can be considered an umbrella term for a collection of individuals with common clinical characteristics who differ in terms of underlying genetic and environmental risk and neuro-biological profiles. This paradigm shift fundamentally changes the research focus of our field. It also changes its translational imperative, from attempts to find the core ADHD deficit to studies that set out to parse the causal heterogeneity of the condition. Consequently, this paradigm shift has the potential to promote more effective treatments tailored and targeted to address the specific underlying causes affecting different individuals with ADHD (Sonuga-Barke, 2020). This, of course places new demands on study design—requiring very large samples and multiple measures of putative neuro-cognitive function across networks.

In the first paper in the special issue, Buitelaar et al., recognizing this “deep” heterogeneity in ADHD, call for a science-driven precision psychiatry approach to assessing and treating ADHD to optimize outcomes for individuals. Their approach focuses on identifying biomarkers that could help partition genetic and pathophysiological heterogeneity and help tailor pharmacological and non-pharmacological interventions. They reviewed the latest ADHD biomarker findings and discussed whether new statistically-based models could help predict and optimize treatment responses and so the disorder course and outcome for people with the condition. Their review was comprehensive, including genetic factors, peripheral metabolomic markers (such as plasma, serum, urine, etc.), and brain markers derived from functional structural imaging techniques (EEG, MRI, NIRS etc.). In addition to such markers from “within” the individual, they examined variations in environmental exposures to social privation, nicotine, and alcohol use during pregnancy and early maltreatment. While emphasizing that intervention biomarker studies are in their infancy, the way the authors highlight this approach's potential to facilitate better treatment will motivate the field to make a significant investment in biomarkers research.

The next two papers dig deeper into different aspects of this precision approach. The first paper discusses, in particular, the potential of genomic and then brain-based markers, as a basis for tailoring treatment to make it more effective at the individual level. In the second paper, Haavik discusses how variations in an individual's genome could, in the future, guide drug therapy. This proposal was built on a review of recent developments in ADHD genetics. While recognizing that the large-scale routine implementation of these approaches remains a considerable way off, he argues for a gradual strategy starting with rare ADHD syndromes with highly penetrant risk genes and well-defined neurometabolic aberrations. For example, he cited the work of Fernandez-Castillo that showed the deficiency of L-2- hydroxyglutarate dehydrogenase (L2HGDH), X-linked creatine transporter deficiency, and HUWE1 mutations presenting ADHD symptoms. Similarly, recently a group of researchers found a single homozygous locus on chromosome 18 affecting N-cadherin function shared by affected individuals in Bedouin pedigree in the south of Israel, diagnosed with ADHD according to the DSM-5 and display severe hyperactive behavior (Halperin et al., 2021). We would like to counsel caution and encourage researchers to ensure that ethical considerations relating to the dangers of the inappropriate use of these early screening technologies for eugenic purposes are fully considered to ensure the life and dignity of people with ADHD.

The third paper by Arnett et al. presented an empirical study of the power of variations in brain activity to predict stimulant response. It highlighted the possibility that these could be used to target treatments more effectively to those who would benefit from them. Their general hypothesis was that methylphenidate would benefit those with more divergent brain activity during attention-demanding tasks. In support of this, they found robust evidence of an attenuated frontal visual odd-ball P300 amplitude of child responders relative to neurotypical children and non-responders. This study could provide an important stimulus for future research.

The second form of heterogeneity we are focusing on in this special issue is captured by the second part of the title of this special issue – **Treat the whole patient, not just their symptoms**. This title reflects the fact that different people, while they may express their ADHD in similar ways, can differ enormously from one another in terms of their co-occurring difficulties (conduct problems, autism, dyslexia, depression, and anxiety) and associated impairment variations (e.g., academic, social, employment, etc.). It has been argued that the patterns of co-occurring difficulties, rather than the core symptoms themselves, create much of the impairment and distress associated with ADHD (Gnanavel et al., 2019; Anbarasan et al., 2022). Such a view aligns with the neurodiversity perspective (Sonuga-Barke and Thapar, 2021). This perspective argues that rather than reducing symptoms of ADHD, treatments should focus on reducing impairment by directly targeting key aspects of daily functioning or using reasonable accommodation to create more supportive environments.

The first paper in the Special Issue to address this issue of the treatment of co-occurring conditions is by Mellahn et al. It focuses on treatment of ADHD is the context of autism spectrum disorder (ASD). The authors describe the range of medications used in a large sample of children with ADHD and/or ASD. Children with ADHD and autism combined had higher rates of anxiety, depression, and ODD than those with just autism or ADHD alone. ADHD or the ADHD + ASD groups took more psychotropic medications than the ASD group. While the ADHD with co-morbid ASD reported the highest rate of non-stimulants, antipsychotics, antidepressants. The second paper in this sub-section, by Yin et al., focused on a feature that occurs so commonly with ADHD that some have called for it to be considered part of the broader ADHD phenotype—sleep disorders. In a convenience sample of 100 unmedicated children the authors looked at the relationship between ADHD and sleep problems as rated by questionnaires. Like previous studies, the authors found a robust link between these sets of difficulties and a strong linear relationship between the severity of ADHD symptoms and sleep problems. In a manner worthy of appreciation, the researchers attached the raw data as a supplementary data. This study reminds us that we should consider individual differences in sleep problems when developing treatment plans.

The third study in this sub-section (Ganjeh et al.) takes a novel approach focusing on developing adolescent mental health problems. It is a large naturalistic longitudinal population-based cohort study looking for the relationship between adolescents' mental health disorders and earlier childhood physical activity. It poses the question of whether physical activity can be protective in relation to later mental health problems. The findings were counterintuitive. Childhood physical activity levels were related to lower levels of ADHD symptoms later on in life during adolescence, extending to a more general pattern of mental health outcomes. The authors argue that giving children the opportunity for physical activity could be valuable clinically as an adjunct to other interventions. We would encourage the authors to explore this hypothesis systematically eventually in RCTs. The final study by Korfmacher et al., compared a form of self-management training against neurofeedback in people with ADHD. They found that both treatment options improved core ADHD symptoms. However, only the self-management treatment resulted in self-concept and quality of life improvements. Thus, they emphasized the added value of self-management training over neurofeedback in the treatment of ADHD.

In this special issue, we bring two under-researched aspects of ADHD translational science together—the need for more precise targeting of specific features and characteristics and, at the same time the need to broaden the focus away from core symptoms to other aspects of functioning. These separate ambitions should jointly inform the next steps of treatment development in ADHD.

## Author contributions

ES-B and SZ wrote the final version. All authors contributed to the writing of the manuscript. All authors contributed to the article and approved the submitted version.

## Conflict of interest

The authors declare that the research was conducted in the absence of any commercial or financial relationships that could be construed as a potential conflict of interest.

## Publisher's note

All claims expressed in this article are solely those of the authors and do not necessarily represent those of their affiliated organizations, or those of the publisher, the editors and the reviewers. Any product that may be evaluated in this article, or claim that may be made by its manufacturer, is not guaranteed or endorsed by the publisher.
